# Non-isotropic noise correlation in PET data reconstructed by FBP but not by OSEM demonstrated using auto-correlation function

**DOI:** 10.1186/1471-2342-5-3

**Published:** 2005-05-13

**Authors:** Pasha Razifar, Mark Lubberink, Harald Schneider, Bengt Långström, Ewert Bengtsson, Mats Bergström

**Affiliations:** 1 Uppsala University, Centre for Image Analysis, Lägerhyddsvägen 3, SE-752 37 Uppsala, Sweden; 2 Uppsala Imanet AB (PET Centre), Uppsala University Hospital, SE-751 85 Uppsala, Sweden; 3 VU University Medical Centre, PET Centre, PO Box 7057, 1007 MB Amsterdam, The Netherlands; 4 Department of Pharmaceutical Biosciences, Uppsala Biomedical Centre, SE-751 24 Uppsala, Sweden

## Abstract

**Background:**

Positron emission tomography (PET) is a powerful imaging technique with the potential of obtaining functional or biochemical information by measuring distribution and kinetics of radiolabelled molecules in a biological system, both *in vitro *and *in vivo*. PET images can be used directly or after kinetic modelling to extract quantitative values of a desired physiological, biochemical or pharmacological entity. Because such images are generally noisy, it is essential to understand how noise affects the derived quantitative values. A pre-requisite for this understanding is that the properties of noise such as variance (magnitude) and texture (correlation) are known.

**Methods:**

In this paper we explored the pattern of noise correlation in experimentally generated PET images, with emphasis on the angular dependence of correlation, using the autocorrelation function (ACF). Experimental PET data were acquired in 2D and 3D acquisition mode and reconstructed by analytical filtered back projection (FBP) and iterative ordered subsets expectation maximisation (OSEM) methods. The 3D data was rebinned to a 2D dataset using FOurier REbinning (FORE) followed by 2D reconstruction using either FBP or OSEM. In synthetic images we compared the ACF results with those from covariance matrix. The results were illustrated as 1D profiles and also visualized as 2D ACF images.

**Results:**

We found that the autocorrelation images from PET data obtained after FBP were not fully rotationally symmetric or isotropic if the object deviated from a uniform cylindrical radioactivity distribution. In contrast, similar autocorrelation images obtained after OSEM reconstruction were isotropic even when the phantom was not circular. Simulations indicated that the noise autocorrelation is non-isotropic in images created by FBP when the level of noise in projections is angularly variable. Comparison between 1D cross profiles on autocorrelation images obtained by FBP reconstruction and covariance matrices produced almost identical results in a simulation study.

**Conclusion:**

With asymmetric radioactivity distribution in PET, reconstruction using FBP, in contrast to OSEM, generates images in which the noise correlation is non-isotropic when the noise magnitude is angular dependent, such as in objects with asymmetric radioactivity distribution. In this respect, iterative reconstruction is superior since it creates isotropic noise correlations in the images.

## Background

Positron Emission Tomography (PET) is a technique based on tracing of molecules labelled with positron-emitting radionuclides to image metabolism, physiology, and functionality in vivo in organs and tissues. PET has become an important, non-invasive technique for providing functional information about specific organs and areas of disease, and is used increasingly in clinical diagnosis, medical research and drug development. One of the most important properties of PET is its ability to supply quantitative values derived from functional images [1].

PET images are usually reconstructed either analytically by filtered back projection (FBP) or iteratively by ordered subsets expectation maximisation (OSEM), a much faster variation of maximum likelihood expectation maximisation (ML-EM) [2]. The former method is based on dividing the raw data into a number of subsets of projections (OS level) followed by applying a standard EM algorithm [2,3].

FBP utilizes the 2D distribution from multi-angular projections [4], projects back these projections after applying 1D convolution with a specific high pass filter [5] to a common image plane. The filter used in FBP consists of a ramp filter, which is used to remove the blurring induced by the back projection. However, the filter also amplifies high-frequency noise. Therefore the filter is usually combined with a low-pass filter, such as a Hanning filter, to reduce noise. The properties of reconstructed images, using various filter functions, have been exploited [6], indicating how different degrees of filtering leads to different appearance and pattern of noise in the images. FBP is a relatively fast process but it has the drawbacks that the generated images contain more noise and are more sensitive to disturbing factors, such as patient movements during and between transmission and emission scans. Different algorithms [7] have been evaluated based on Fourier analysis to shorten reconstruction times and improve signal-to-noise ratio at equivalent resolution.

In traditional PET scanners, with rotating ^68^Ge/^68^Ga transmission sources, the main sources of noise are in decreasing order of magnitude: emission, transmission and blank scans [8]. With newer attenuation correction modes, e.g. CT, the noise from emission is clearly dominating. Detectors and the recording system in the tomograph affect two characteristics of noise: magnitude and texture. The detector system affects only the noise magnitude, whereas the recording system affects both the noise magnitude and texture [9]. The choice of reconstruction algorithm and type of convolution kernel used in the reconstruction algorithm significantly affects the magnitude and correlation of noise [10].

Another important factor related to noise that affects the quality in PET images, and even more the potential to estimate precision in a measurement, is the correlation of noise between pixels. Image quality, in its simplest form characterized by pixel signal-to-noise ratio [11], becomes an inadequate measurement when different types of noise correlation exist between the pixels within the images. It has been shown that 3D PET images contain strong correlation between the values in adjacent pixels and the correlation is found to be a complex function [12]. The correlation of each element influences one or two pixels of the nearest neighbours [13].

The noise properties of emission tomographic images reconstructed by ML-EM and FBP have been compared [14]. The comparisons were based on the covariance matrix and noise properties as a function of iteration for ML-EM and as a function of noise apodization filter for FBP. The covariance matrix gave information about noise magnitude and texture, and indicated differences in noise pattern depending on applied reconstruction algorithm. Further studies have indicated that low intensity regions of images reconstructed by iterative algorithms tend to have low noise or a local noise pattern. In contrast, images reconstructed by FBP tend to have a much more globally distributed noise pattern [15,16].

Comparisons have been made between images acquired in both 2D and 3D modes and in synthetic images. Due to differences in axial resolution and noise correlation between the two modes we compared images acquired in both modes. The decrease in axial resolution in 3D is attributed to the retraction of septa leading to increase in the crystal solid angles with a broadening of the point spread function and the smoothing effect introduced by 3D reconstruction. The observed 3D images are noisier near the centre of the FOV yet the axial correlation is lower in 2D images [17].

An essential aspect of PET is its ability to obtain quantitative values for regions of interest (ROIs) within the images. These values by themselves can have diagnostic value, which can give insights into physiology of normal and diseased tissues or can give important information about drug distribution or interaction with target systems. Since PET images are inherently noisy, the standard method to reduce noise for the quantitative estimates is to take averages over several pixels within an ROI. This is an adequate method, but because of the correlation between the pixels, it is not trivial to assign a precision value to these averages. Moreover an understanding of the noise properties in PET images is essential for adequate use of parametric images with statistical criteria included, such as in studies of blood flow changes during behavioural paradigms. Furthermore, it has been shown that variable noise levels in the different PET images, dramatically affect the subsequent principal component analysis, unless they are properly handled [18].

Although different aspects of noise have been covered extensively in the literature, we still feel that one aspect has not been adequately covered: the angular dependence of noise correlation in cases when the investigated object is asymmetrical. With asymmetric objects, the count rates will be different in the different acquisition angles and the relative magnitude of the noise is therefore different. It is possible that this angular dependent noise would during the image reconstruction propagate to the images and there generate a noise correlation which is non-isotropic.

Here our main focus was to demonstrate the relationship between the shape of the experimental object and the properties of noise, especially the angular dependence of correlation in the PET images. Comparisons were made between images acquired in both 2D and 3D modes reconstructed using FBP and OSEM. We also compared results from autocorrelation function and the results using a covariance matrix applied to synthetic images.

## Methods

All experiments were performed on an ECAT Exact HR+ [19]. This scanner contains 32 detector rings separated by removable septa and is capable of performing 2D and 3D data acquisition. The total number of bismuth germanate detectors is 18 432 generating 63 contiguous image planes in the form of [128 × 128] matrices with an axial field of view (FOV) of 155 mm. These experiments were performed using either a 20-cm-diameter, 20-cm-long water-filled cylindrical phantom [20,21], an elliptical torso phantom (long axis 30 cm, short axis 20 cm), or a combination of the 20-cm phantom with two adjacent 5-cm diameter, 20-cm-long water-filled cylinders positioned on opposite sides of the larger phantom (satellite phantom) to create a variety of noise textures in the images.

Two different radionuclides, ^18^F and ^68^Ga, with 110 min and 68 min half-life, respectively, were used. ^18^F was produced using a Scanditronix MC-17 cyclotron (Scanditronix AB, Uppsala, Sweden). ^68^Ga was obtained from a ^68^Ge generator [22].

Prior to each experiment, a blank scan with rotating ^68^Ge /^68^Ga rod sources was acquired. The phantom was filled with 60 MBq of either ^18^F or ^68^Ga, placed at the centre of the FOV, and 30-min emission scans were made in both 2D and 3D mode. Finally to avoid artefacts in the images caused by movement of the object between transmission and emission scan, a 10-min post-injection ('hot') transmission scan was performed. A segmentation technique, as included in the ECAT 7.2 software (CTI, Knoxville, Tennessee) was applied on the hot transmission data before it was used for attenuation correction in the reconstruction process. The radioactivity concentration and mode of attenuation correction correlated reasonably well with the conditions used in clinical scans.

For reconstruction of raw PET data, both FBP and OSEM which were included in the scanner software were used for reconstructing the data. The 3D data is rebinned to a 2D dataset using FORE followed by 2D reconstruction using either FBP or OSEM. Different types of low pass filters were used with each of the reconstruction methods, e.g. 4 mm (FWHM) Hanning and 6 mm (FWHM) Gauss filter. For FBP the filtering was made as part of the reconstruction ramp filter in the projections. For the iterative reconstruction it was made as post-reconstruction smoothing filters. The use of the same filter with FBP and OSEM ensured similar spatial resolution.

In a simulation study on images reconstructed using FBP, a program was developed. For generating the synthetic PET images, Matlab (The Mathworks, Natick, Massachusetts) was used in which sinograms of a cylindrical phantom were calculated by forward projection of noise-free synthetic images of this phantom. An additive, Poisson noise with different magnitude in different angles was added to the sinograms, and images were reconstructed with FBP using Matlab's "iradon.m" routine.

Another program was developed, using Matlab to calculate the ACF of the reconstructed PET images, performed both in the frequency and spatial domain. Papoulis [23] describes the autocorrelation function as a function often used in exploring similarity between images or image parts. The autocorrelation function *A*_*ff *_of a random process *f *is defined as a mean of the product of the random variables *f *(*x*_1_, *y*_1_, *w*_*i*_), and *f *(*x*_2_, *y*_2_, *w*_*i*_),

*A*_*ff *_(*x*_1_, *y*_1_, *w*_*i*_, *x*_2_, *y*_2_, *w*_*i*_) = *E *{*f *(*x*_1_, *y*_1_, *w*_*i*_) *f *(*x*_2_, *y*_2_, *w*_*i*_)}   (1)

where *E *is the mathematical expectation operator, *f *(*x*, *y*, *w*_*i *_) is a stochastic or random process, and *w*_*i *_is an element for a set of all events. Here we created the ACF based on the following equation by Kay [24].

*r*_*yy *_[*t*, *l*] = *r*_*xx *_[*t*] *r*_*zz *_[*l*]   (2)

where *r*_*xx *_[*t*] and *r*_*zz *_[*l*] are valid 1D ACFs.

The spatial equation is based on 2D cross-correlation of the

matrix *a*_*i, j *_with resolution of *i *× *j *with itself using the lags k and l



where k and l refer to lags of the function and

max(1, 1 - *k*) ≤ *i *≤ min(*m*, *m *- *k*)

and

max(1, 1 - *l*) ≤ *j *≤ min(*n*, *n *- *l*)

A number of central slices of the image set were read to avoid effects at the edges of the FOV. The level of the noise is slightly higher near the centre of the FOV for 3D but not 2D mode. Subsequently, a matrix containing 25 × 25 pixels from the central part of the image was selected as a mask for the ACF. After subtraction of the average over this matrix, an ACF image was generated in which each pixel was set to the product of the mask matrix and a matrix in the original image, centred around the selected pixel position. The ACF image was then normalized by dividing each pixel value by the maximum pixel value within the ACF image. The results from this procedure applied to images from all experiments were studied and compared.

The aim of this application was to study the noise correlation between the pixels within each image. The method was to analyse the shape of the 2D autocorrelation function in the images. The program results in images that can then be used to visualize and compare the ACF from PET images obtained with different reconstruction algorithms and different acquisition modes. 1D vertical and horizontal profiles through the ACF images were plotted to illustrate noise correlation.

Third program was developed to calculate the covariance matrices based on a matrix with 25 × 25 pixels from the central part of synthetic reconstructed images using the FBP method, with the level of noise in projections angularly variable. This study was performed on 1500 synthetic images repeated 10 times, with independent random generated noise and the result as covariance matrices averaged. 1D covariance values were calculated using the equation,



where



N is the number of 2D synthetic images, which are 1500 in each attempt and  refers to the mean value of column vector containing all centre pixels *x*_*i *_of all synthetic images.  refers to the mean value of the column vector containing all j^th ^adjacent pixels from the centre pixel *x*_*i *_of all synthetic images.

The aim of this application was to study whether the autocorrelation and covariance methods gave identical results for noise magnitude and texture. To simplify this comparison of noise properties, we plotted a 1D profile through the middle section of the result after applying ACF and the result from calculated covariance values between same pixels.

## Results

**Figure 1:**
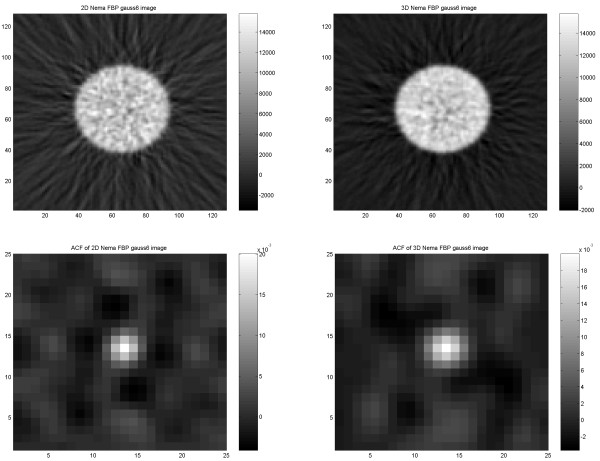
Results of the cylindrical NEMA phantom study. 2D acquisition image (upper left) and 3D acquisition image (upper right) reconstructed using FBP with applied 6 mm (FWHM) Gaussian filter. Corresponding ACF images of 2D (lower left) and 3D image (lower right).

**Figure 2:**
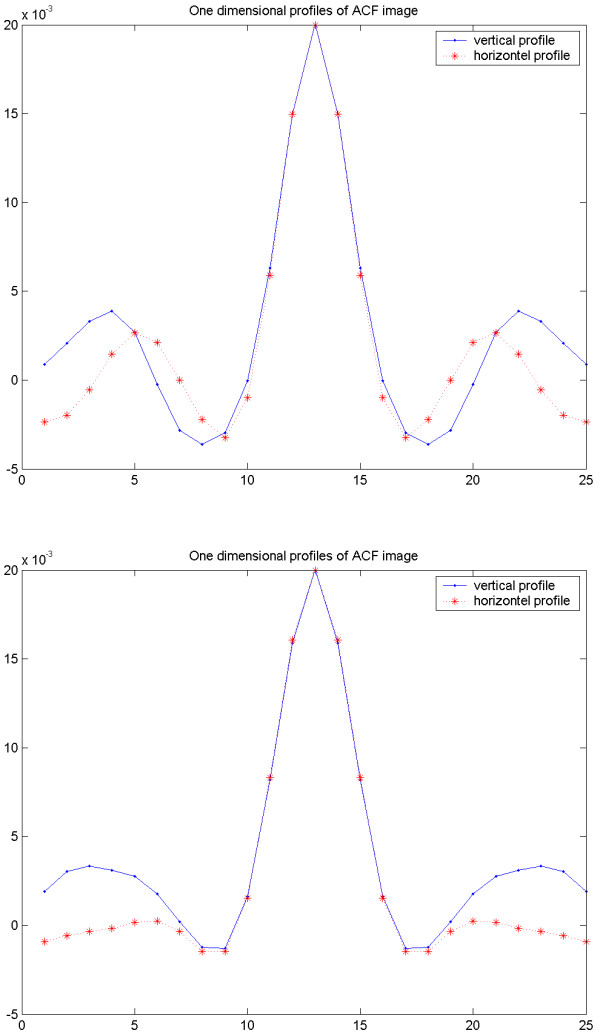
Results of the cylindrical NEMA phantom study. Vertical (dash point) and horizontal profile (dash star) through the centre of the ACF image from 2D (upper) and from 3D acquisition image (lower) reconstructed using FBP with 6 mm Gaussian filter.

**Figure 3:**
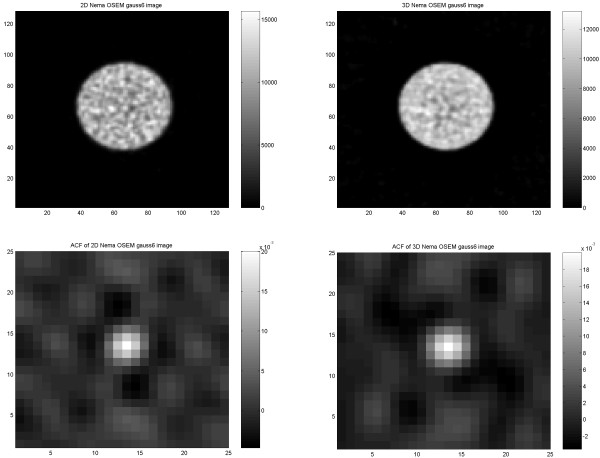
Results of the cylindrical NEMA phantom study. 2D image (upper left) and 3D image (upper right) reconstructed using OSEM with applied 6 mm (FWHM) Gaussian filter. Corresponding ACF images of 2D (lower left) and 3D image (lower right).

**Figure 4:**
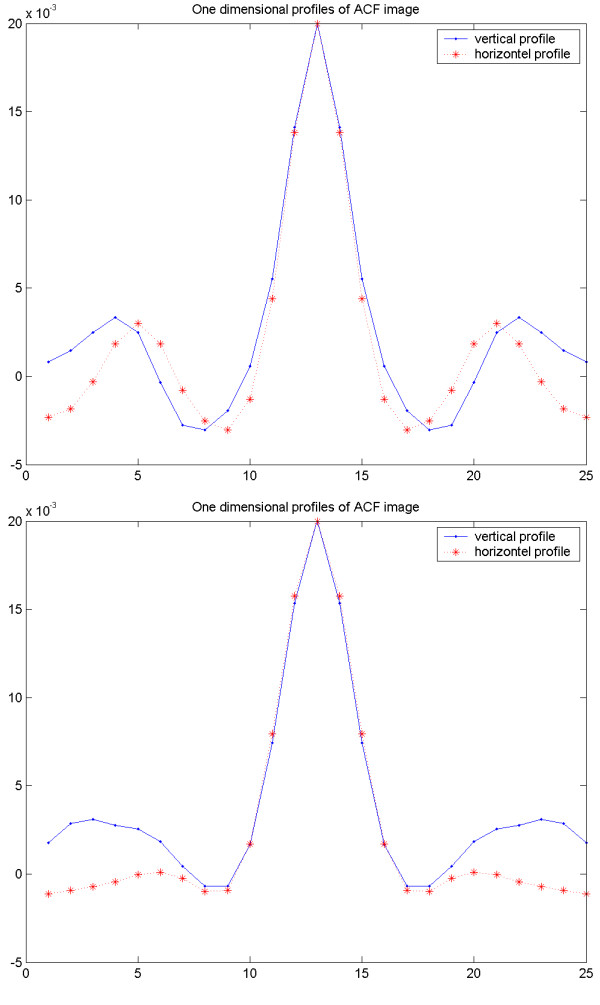
Results of the cylindrical NEMA phantom study. Vertical (dash point) and horizontal profile (dash star) through the centre of the ACF image from 2D (upper) and 3D acquisitions (lower) reconstructed using OSEM with 6 mm Gaussian filter.

In the 2D study on the NEMA phantom (Figures 1, 2, 3, 4), the results indicate an identical and isotropic form with a similar pattern of noise texture independent of applied reconstruction methodology and used filter (6 mm Gaussian and 4 mm Hanning). In the 3D study the same behaviour was noticed independent of applied reconstruction method and used filter. Image noise, propagating into the ACF images, generates low frequency oscillations.

**Figure 5:**
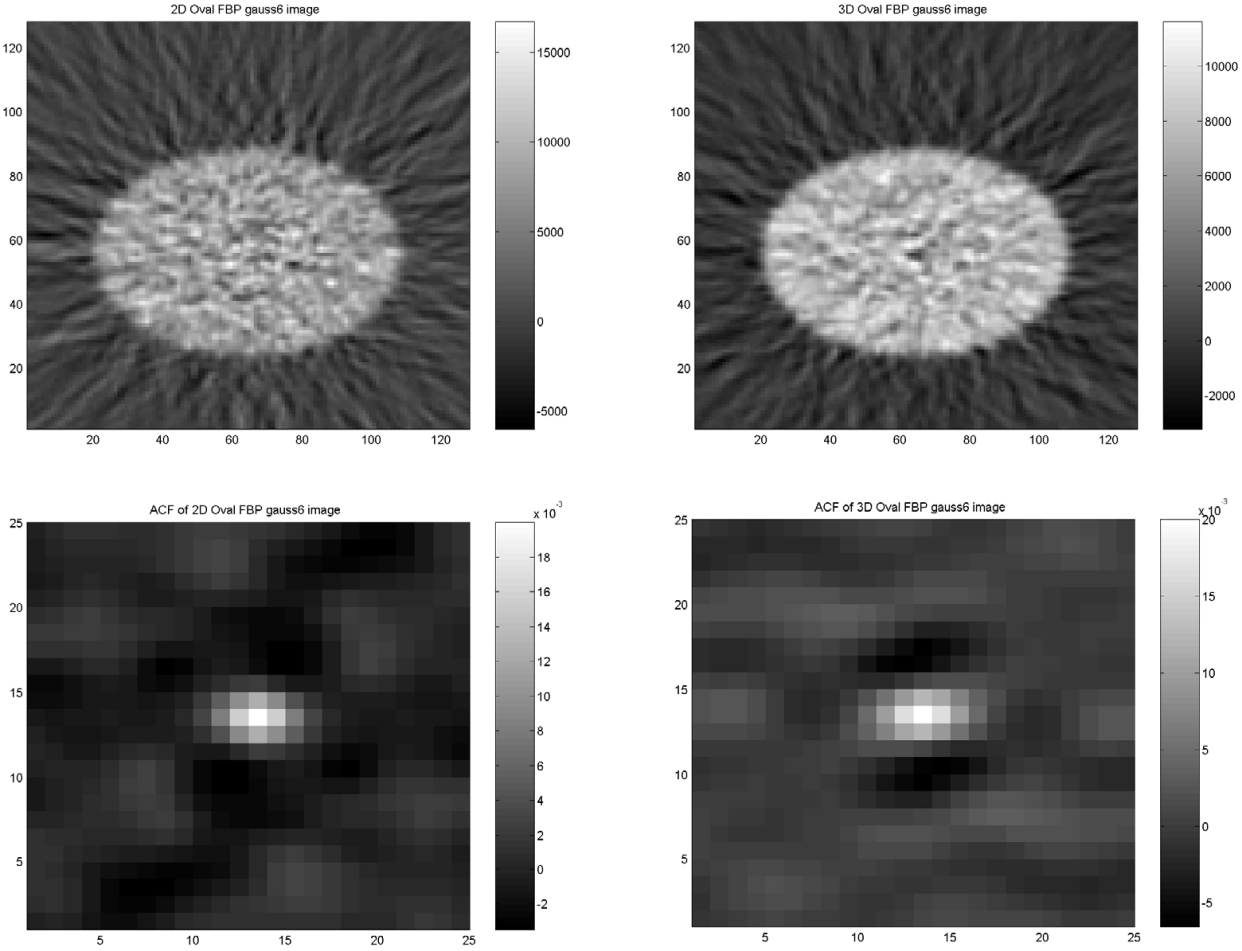
Results of the elliptical torso phantom study. 2D image (upper left) and 3D image (upper right) reconstructed using FBP with applied 6 mm (FWHM) Gaussian filter. Corresponding ACF images of 2D (lower left) and 3D image (lower right).

**Figure 6:**
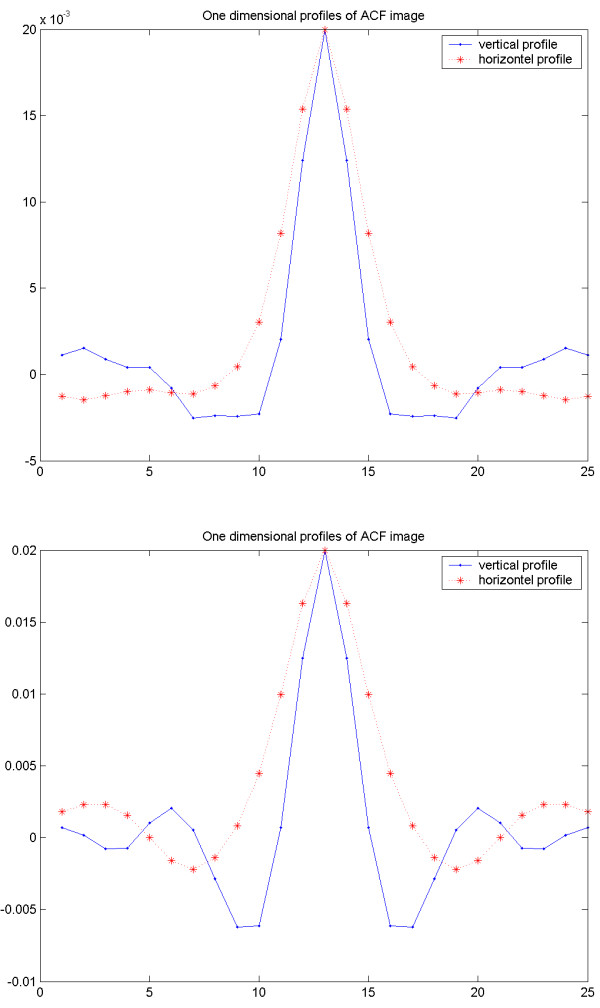
Results of the elliptical torso phantom study. Vertical (dash point) and horizontal profile (dash star) through the centre of the ACF image obtained with 2D (upper) and 3D acquisition (lower) reconstructed using FBP with 6 mm Gaussian filter.

**Figure 7:**
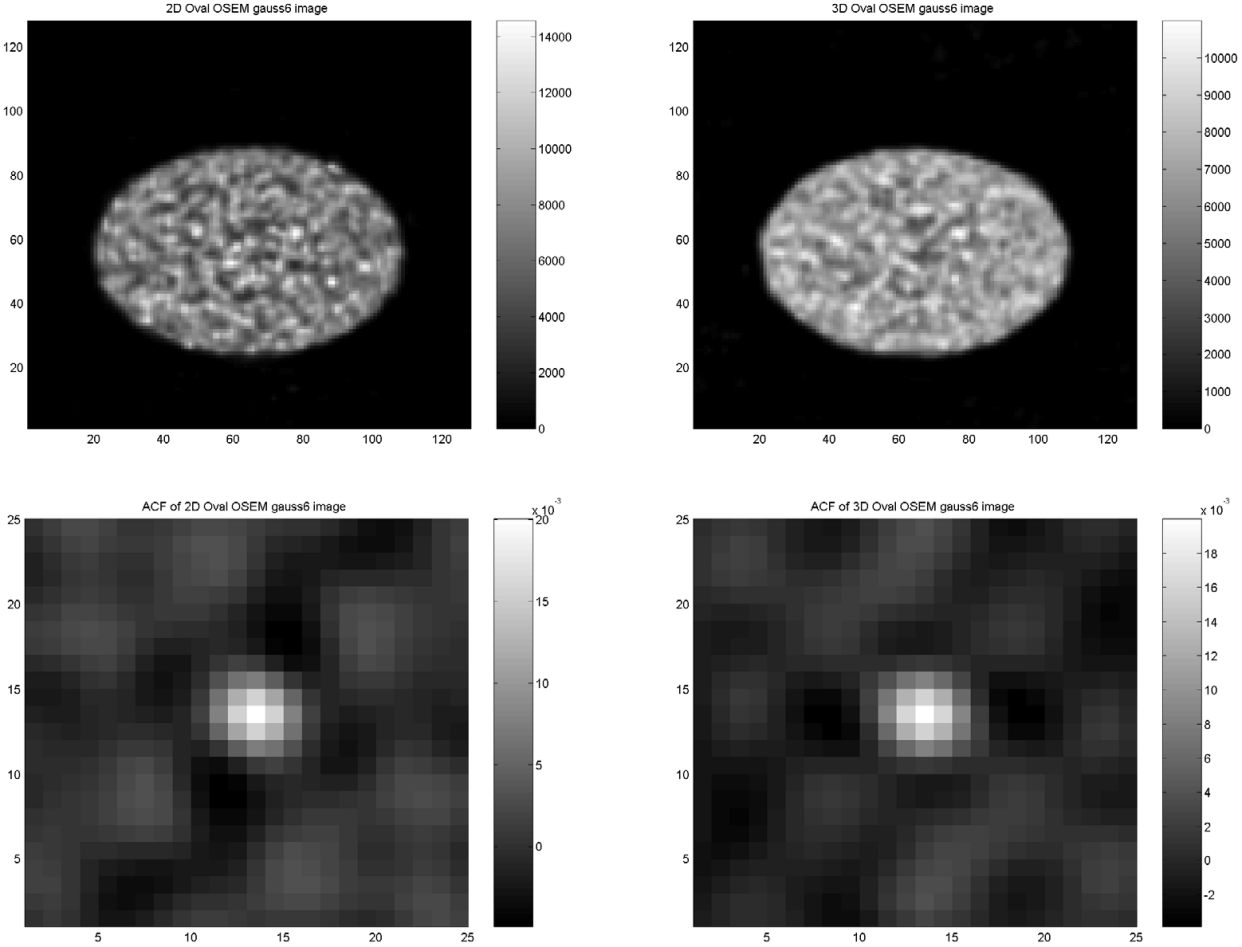
Results of the elliptical torso phantom study. 2D image (upper left) and 3D image (upper right) reconstructed using OSEM with applied 6 mm (FWHM) Gaussian filter. Corresponding ACF images of 2D (lower left) and 3D image (lower right).

**Figure 8:**
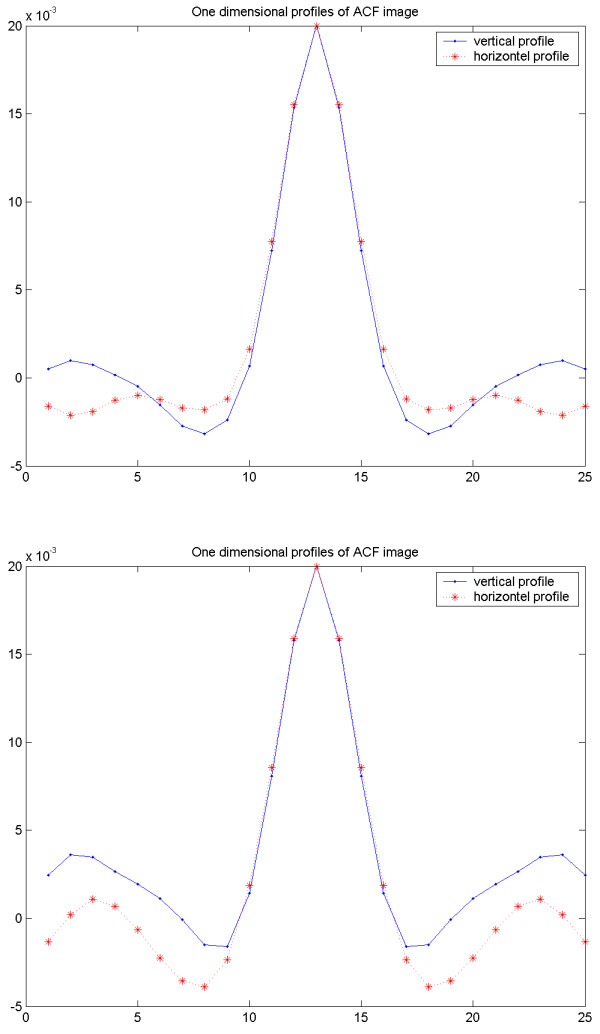
Results of the elliptical torso phantom study. Vertical (dash point) and horizontal profile (dash star) through the centre of the ACF image obtained with 2D (upper) or 3D acquisition (lower) reconstructed using OSEM with 6 mm Gaussian filter.

In both the 2D and 3D study on the torso phantom (Figures 5, 6, 7, 8), the results indicate non-isotropic behaviour of the noise with a dissimilar pattern of noise texture on images reconstructed using FBP, independent of used filter (6 mm Gaussian and 4 mm Hanning). However, the results from images reconstructed using OSEM indicate an identical and isotropic form with a similar pattern of noise texture in both 2D and 3D and independent of used filter.

**Figure 9:**
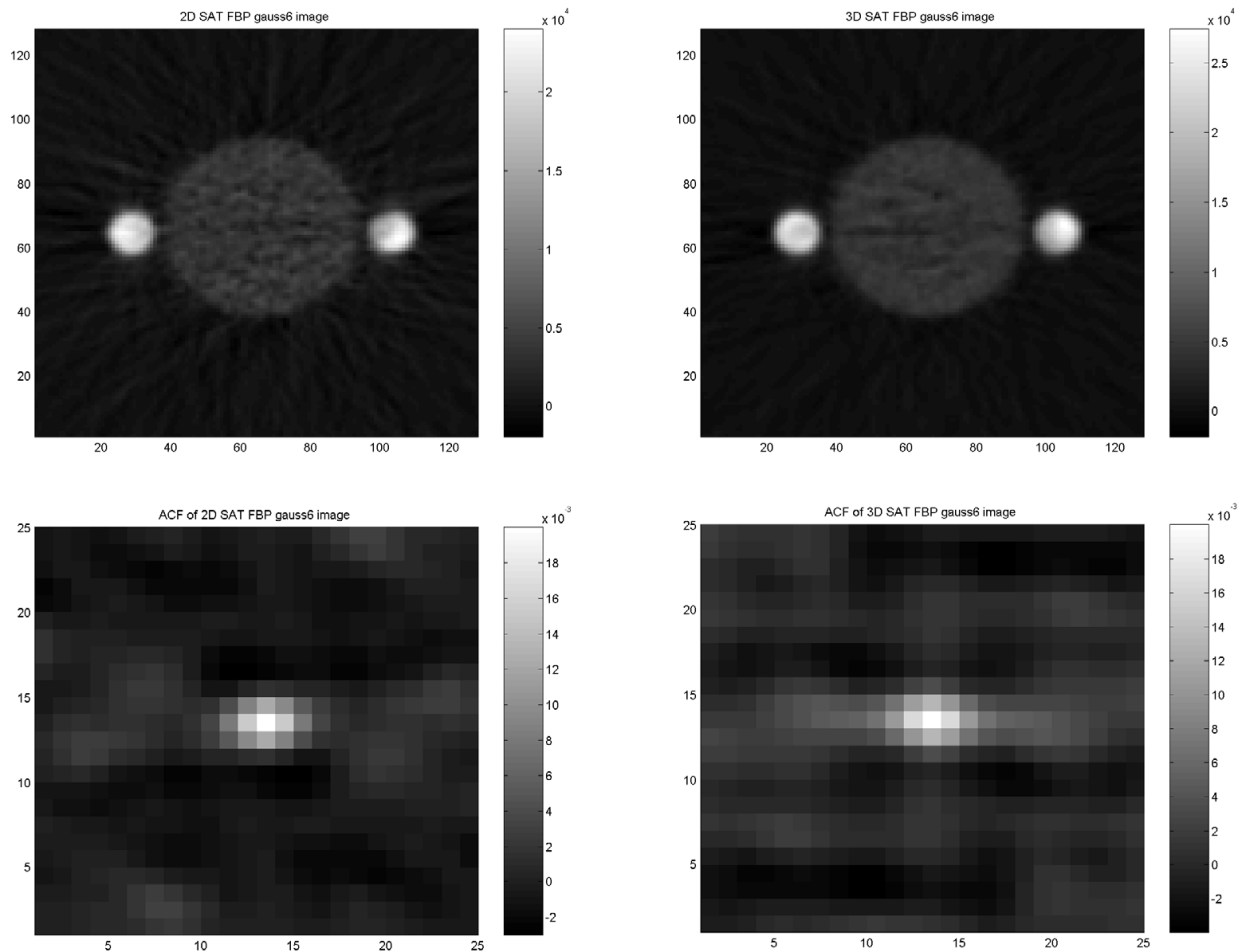
Results of the satellite phantom study (NEMA phantom with adjacent hot cylinders). 2D image (upper left) and 3D image (upper right) reconstructed using FBP with applied 6 mm (FWHM) Gaussian filter. Corresponding ACF images of 2D (lower left) and 3D image (lower right).

**Figure 10:**
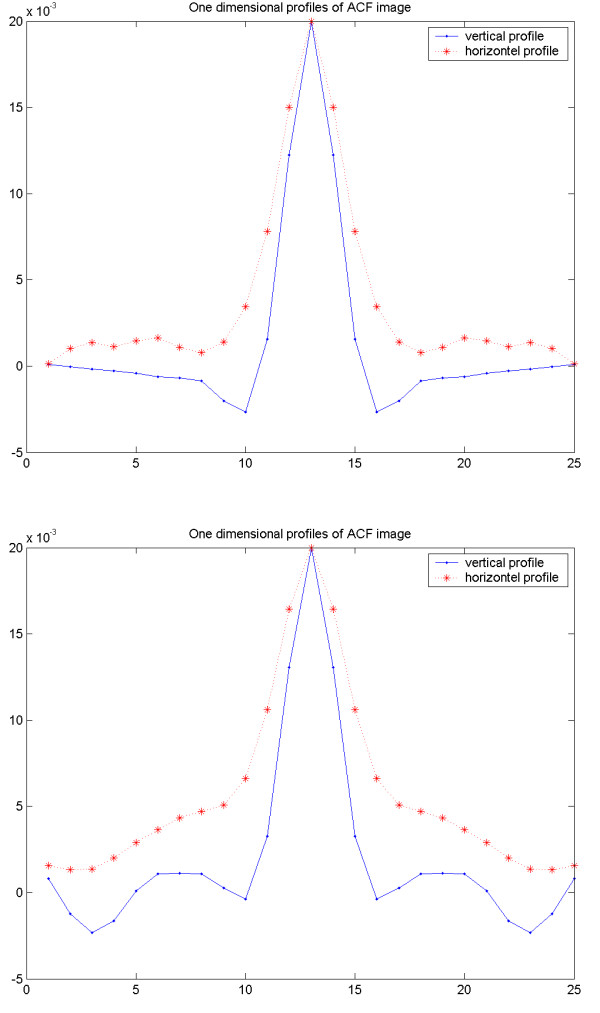
Results of the satellite phantom study. Vertical (dash point) and horizontal profile (dash star) through the centre of the ACF image obtained with 2D (upper) and 3D acquisition (lower) reconstructed using FBP with 6 mm Gaussian filter.

**Figure 11:**
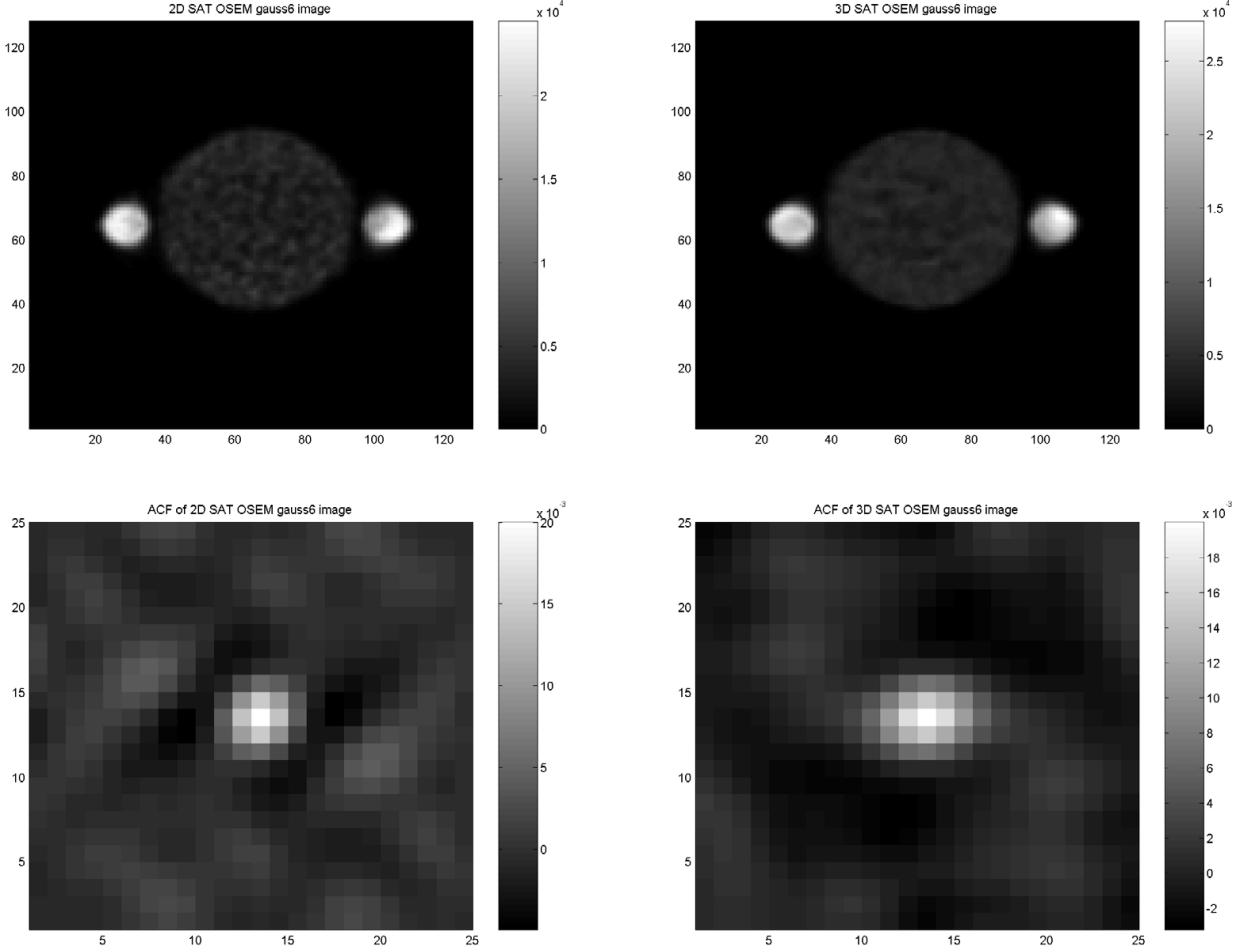
Results of the satellite phantom study (NEMA phantom with adjacent hot cylinders). 2D image (upper left) and 3D image (upper right) reconstructed using OSEM with applied 6 mm (FWHM) Gaussian filter. Corresponding ACF images of 2D (lower left) and 3D image (lower right).

**Figure 12:**
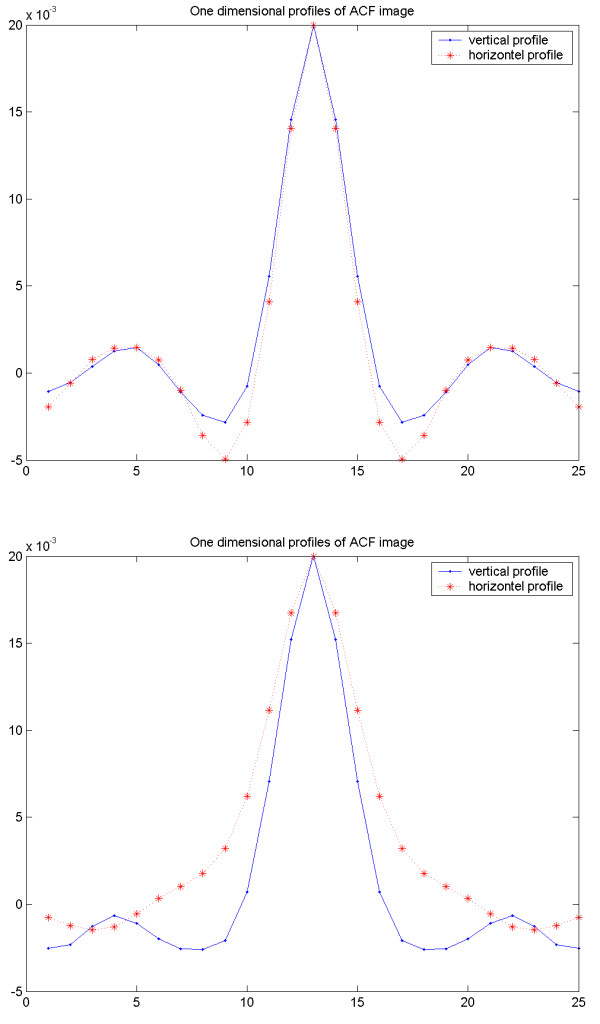
Results of the satellite phantom study. Vertical (dash point) and horizontal profile (dash star) through the centre of the ACF image obtained with 2D (upper) and 3D acquisitions (lower) reconstructed using OSEM with 6 mm Gaussian filter.

The main reason for performing the satellite phantom study was to observe how FBP and OSEM would handle an image with a stronger variation of the noise-texture. In the study performed on the satellite phantom, similar results were observed as in the study with the elliptical phantom. Hence the FBP generated a non-isotropic noise correlation whereas OSEM generated much more rotational symmetric pattern, although not fully for the 3D acquisition (Figures 9, 10, 11, 12).

**Figure 13:**
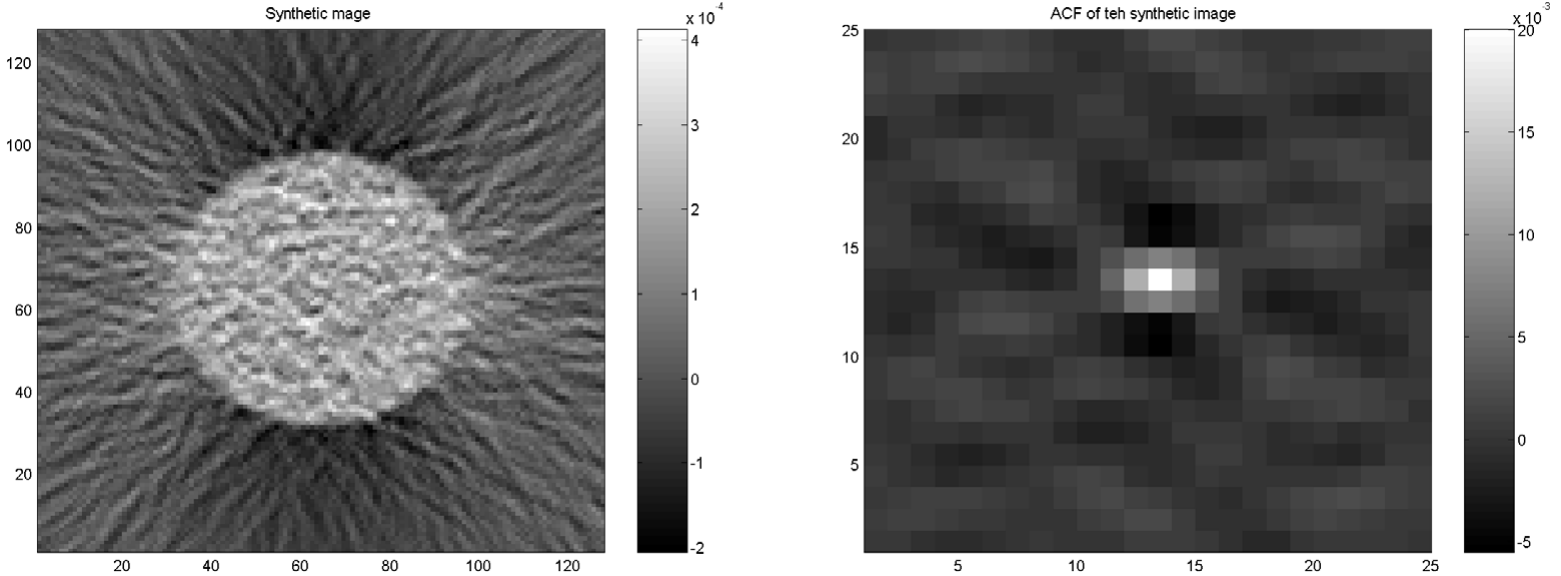
Example of synthetic image with additional Poisson noise with an angular dependent magnitude (left) and the result of applying ACF on the images (right).

**Figure 14:**
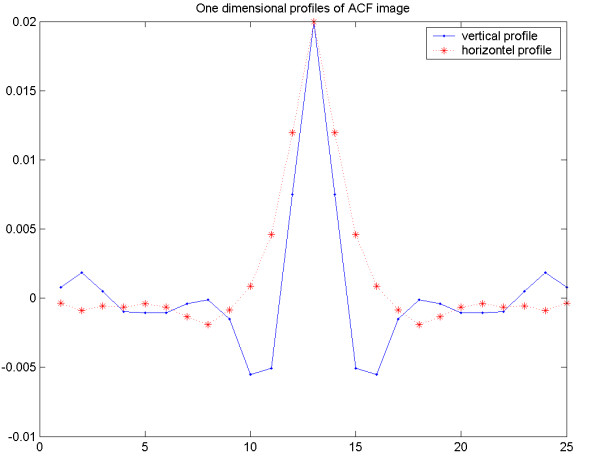
Vertical (dash point) and horizontal profile (dash star) through the centre of the ACF image obtained from the synthetic PET image

The ACF was applied to study noise correlation in synthetic images in which Poisson noise with different magnitude was added to the different projections (Figure 13). 1D profiles through the ACF image indicate a non-isotropic behaviour of the noise correlation (Figure 14). This study shows that FBP generates images in which the noise correlation is non-isotropic when the noise magnitude is angular dependent, such as in objects with asymmetric radioactivity distribution.

**Figure 15:**
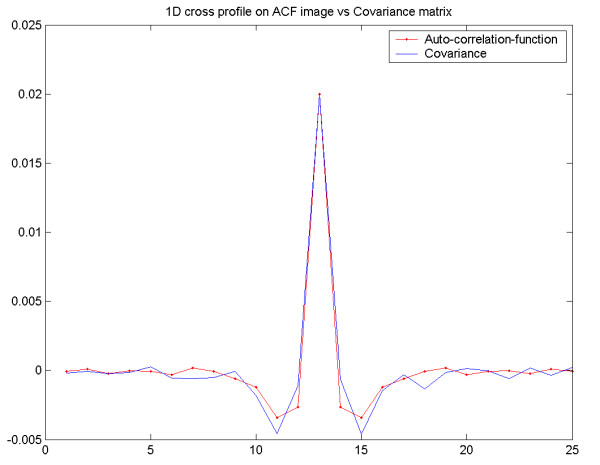
Shows 1D profile through the centre of ACF (dash dot curve) and covariance matrix (solid curve) from the study using synthetic images.

Figure 15 illustrates the result comparing a 1D profile using the 2D ACF and averaged covariance matrix from the study in synthetic images. The results are almost identical concerning noise correlation. In this case a higher resolution ramp filter was used.

## Discussion

The aim of the present study was to explore the properties of noise correlation in PET images. We expected that there could be differences in noise correlation in different directions when the noise level differed in different angular projections during acquisition. This would be the case with elliptical phantoms and under conditions where the radioactivity concentration was non-uniform. We therefore generated three sets of phantoms for the experimental determination of noise correlations. To explore noise correlation and to illustrate its features, we developed a program for the generation of auto-correlation images.

Applying the auto-correlation function on the reconstructed PET images shows a very good similarity vs. dissimilarity concerning noise behaviour between two of the most commonly used reconstruction methodologies. ACF images from PET images reconstructed by FBP showed that the correlation pattern becomes asymmetrical when a study is performed on a non-circular phantom. This is illustrated in Fig. 5 and 6 for the elliptic phantom and Fig. 9 and 10 for the "satellite phantom". This is due to the way noise is handled in the FBP algorithm where the noise reduction or amplification by the filtering is the same in all projections. The back projection will then distribute different noise levels in different angular directions. On the other hand, the ACF image obtained from PET data reconstructed using OSEM did not show such a tendency. The pattern of correlation was shown to be isotropic and independent of the shape of the studied phantom. One possible explanation for this feature of iterative reconstruction is that the technique inherently attempts to iterate to similar deviations for each angular projection, a process that tends to equalise noise for the different projections. These results suggest the need for further studies on OSEM. We also expected that the result from applying autocorrelation function on PET data would provide the same information as calculating covariance matrix. Using ACF and covariance matrix to study synthetic images gave almost identical results for noise correlation and its behaviour.

An advantage of using ACF instead of covariance or correlation is that by applying ACF on an image one can study and explore how adjacent, surrounding pixels affect the middle pixel, and illustrate the result in an image which can be visually inspected and used for further conclusions. E.g. artefacts due to detector or electronics mismatch might be easily revealed. Another advantage of applying ACF is that the function can be applied on one or a small number of images.

A limitation of using ACF for a noise correlation description is that the method is only applicable on images without structural information, i.e., images obtained from uniform objects. It is not feasible to obtain reliable results from images with structural information, e.g. an image of human brain, because the data from the structural part affect the results. Another limitation of using ACF is that the function is sensitive to data with non-stationary statistics, e.g. variance, and consequently the mask used for performing ACF cannot be large. It is well known that the noise variance is variable over the image field but this aspect can be minimised by using a small mask. Here we selected to use a central mask of 25*25 pixels, knowing that in this central region the noise amplitude is perhaps constant.

## Conclusion

We conclude that the noise correlation in FBP is angular and object dependent, and therefore it is e.g. not possible to apply general statistical methods to estimate precision in an average over a region of interest. With iterative reconstruction, the noise correlations seem to be more symmetric and vary less with the object. It might therefore be possible to apply generalised statistical methods but with due consideration to the fact that there is a significant correlation between pixels.

## Competing interests

The author(s) declare that they have no competing interests.

## Authors' contributions

Authors PR and MB helped with the design of the study. They created the method for applying ACF and performed the image and data analysis and drafted the manuscript.

Author ML helped run the camera, acquire and reconstruct the data and write this paper.

Authors HS, EB and BL helped with some of the practical approaches and the writing of the paper.

## Pre-publication history

The pre-publication history for this paper can be accessed here:
